# The Differing Phenotypes of the Three Most Common Postsynaptic Congenital Myasthenic Syndromes Governed by Their Underlying Molecular Pathogenic Mechanisms

**DOI:** 10.1002/mus.70127

**Published:** 2026-07-01

**Authors:** David Beeson

**Affiliations:** ^1^ Neurosciences Group, Department of Clinical Neuroscience University of Oxford Weatherall Institute of Molecular Medicine Oxford UK

**Keywords:** AChR deficiency, congenital myasthenic syndrome, DOK7, phenotype, RAPSN

## Abstract

The congenital myasthenic syndromes are rare disorders of impaired signal transmission at the neuromuscular junction. Despite next generation sequencing facilitating the identification of variants in myasthenic‐associated genes, these variants are frequently of unknown significance and the clinical diagnosis can be delayed. It is mutations in the genes encoding neuromuscular junction proteins AChR, RAPSN, and DOK7 that underlie most cases. Appreciation of key phenotypic features that distinguish between AChR deficiency due to mutations in *CHRNE*, AChR deficiency due to mutations in *RAPSN,* and the “limb girdle” myasthenic syndrome due to mutations in *DOK7* can facilitate early diagnosis. While severity for each of these conditions is markedly variable and thus there are exceptions to the rule, the *CHRNE* mutations result in stable generalized fatigable weakness with marked ophthalmoparesis, *RAPSN* mutations often result in respiratory crises in neonates or early childhood that tend to resolve with age, and *DOK7* mutations cause a limb girdle myasthenic pattern of weakness that may present at birth or shortly after the walking motor milestone is achieved, is slowly progressive but responds well to treatment. The differences and time course for these phenotypes may be explained by the underlying molecular mechanisms. For the *CHRNE* mutations, patients at least partially function on the fetal form of the AChR; for *RAPSN* mutations, AChR clustering on the postsynaptic membrane is destabilized; and for *DOK7* mutations, the stability of endplate structures is affected. Each of these disorders responds to treatments that can be life‐transforming and so early recognition is key.

## 1 Introduction

**FIGURE 1:**
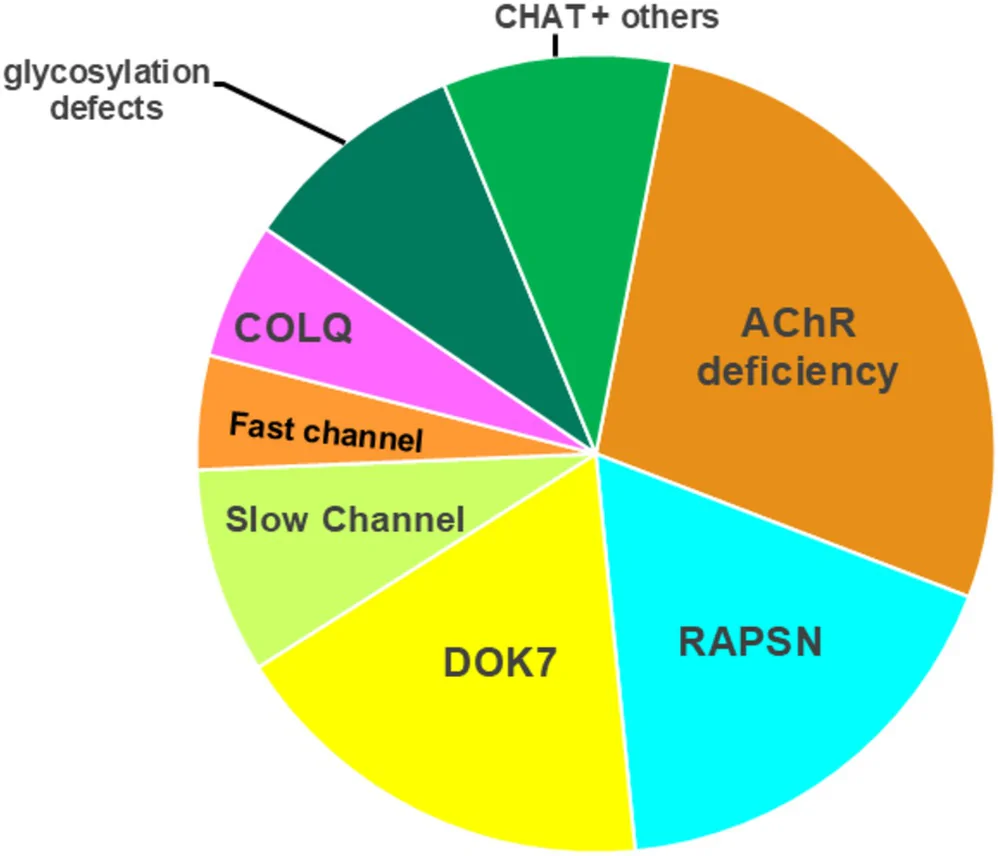
Pie chart of major CMS subtypes for over 600 patients where genetics has been studies in Oxford demonstrating the frequency of CHRNE deficiency, RAPSN, and DOK7 mutations.

For ease of classification the congenital myasthenic syndromes (CMS) can be broadly grouped according to the location of the defective neuromuscular junction protein and thus are often classed as presynaptic, synaptic and postsynaptic. Diagnosis depends upon clinical suspicion, electrophysiological tests, and increasingly, on identification of specific gene variants and functional analysis of their effect. Next generation sequencing (NGS) has recently helped to identify new CMS causative genes which may also be expressed in non‐muscle tissues or even ubiquitously expressed, showing that protein defects not specifically confined to the neuromuscular junction (NMJ) can cause myasthenia [1, 2]. In many of the more recently identified CMS‐associated genes aberrant neuromuscular transmission is only one component of a more complex phenotype in which muscle, the central nervous system and other organs may also be affected [1, 2, 3, 4, 5, 6, 7]. However, CMS due to mutations in the neuromuscular junction postsynaptic proteins are by far the most common, with variants in the AChR epsilon subunit (CHRNE), rapsyn (RAPSN) and downstream of kinase‐7 (DOK7) dominating (Figure 1) [1, 2, 8] There are a variety of molecular mechanisms that can affect efficient functioning of the muscle AChR, such as mutations causing slow channel syndromes, fast channel syndromes or reduced conductance, but for ease of comparisons with RAPSN and DOK7 CMS this short review will only cover the AChR mutations in *CHRNE* that cause deficiency of endplate AChR.

## 2 The Developmental Profile of the Human AChR and Neuromuscular Synapse

The temporal expression of the muscle AChR isoforms and maturation processes for the neuromuscular junction are crucial in determining the phenotypes of the differing forms of CMS.

Muscle AChRs are the archetypal cys‐loop ligand gated ion channel. They are glycosylated transmembrane ion channels that mediate synaptic transmission. They are allosteric (existing in several different conformations) and the binding of two ACh to each receptor favors a conformational change that results in a brief activation of the channel and the influx of cations. Each AChR is made up of five subunits arranged in a pentameric structure around the central ion pore (for more detail see the recent cryo‐EM studies of the human AChR that reveal the structures at the molecular level [9, 10]). In mammalian muscle there are two types of AChR, a form mainly in fetal muscle that consists of α_2_βγδ and an adult muscle form α_2_βδε [11]. The subunits are homologous, vary in size from 437 to 495 amino acids (once the signal peptide sequence has been cleaved off), and are encoded by separate genes of between 10 and 12 exons. In embryonic muscle, before innervation, fetal AChR are distributed along the length of the muscle fibers. During innervation the AChR are clustered on the postsynaptic membrane and are lost from extrasynaptic sites. As the synaptic structure matures, expression of the γ‐subunit (fetal) mRNA is repressed and is replaced by ε‐subunits (adult) mRNA transcribed from subsynaptic nuclei. In humans, ε‐subunits gradually replace the γ‐subunits within the AChR at the endplates during the third trimester, but by contrast with mice where expression of the γ‐subunit is completely turned off by 3 weeks after birth, in humans the γ‐subunit continues to be expressed at low levels in adult muscle [12].

The switch from γ‐subunit to ε‐subunit expression is part of the maturation of the neuromuscular synapse into a complex structure that facilitates efficient signal transmission.

The classic view of the development of the neuromuscular synapse derives from a series of experiments in mice in which components of the neuromuscular junction were “knocked out” [13]. These experiments revealed what is now known as the AGRN‐LRP4‐MUSK‐DOK7 AChR clustering pathway. Details of this pathway will be discussed in greater depth elsewhere in this review, but, in brief, knockout experiments have highlighted a process in which agrin, released from the motor nerve terminal, binds to LRP4 on the postsynaptic membrane, which in turn interacts with and activates MuSK, a receptor tyrosine kinase, which in turn activates a downstream pathway in which the AChR β‐subunit is phosphorylated, and RAPSN acts to cluster and stabilize the AChR on the postsynaptic membrane [14]. The MuSK signaling is stimulated and amplified by intracellular interaction with DOK7. DOK7 binds specifically to MuSK and when expressed in cultured C2C12 cell‐line myotubes is able to induce large AChR clusters in the absence of neural agrin. As well as stimulating AChR cluster formation these proteins are integrally involved in the formation and stabilization of endplate synaptic structure [15, 16, 17].

As stated above, much of our knowledge of the neuromuscular junction is derived from studies of mice, but surprisingly in CMS when the human mutations are recreated at the equivalent positions in mice for CHRNE, RAPSN, or DOK7 they lead to markedly different phenotypes (Table 1). This likely reflects altered expression profiles and compensatory plasticity of key molecules at the human neuromuscular junction that are not present in mice. The result is survival with a relatively mild phenotype for many patients with CMS that does not occur for the orthologous situation in mice. It also suggests that caution should be applied when extrapolating the efficacy of potential new treatments to humans (whether positive or negative) from preclinical studies in mice.

**TABLE 1:** Mouse neuromuscular junction disease models rarely provide a good model for the human condition.

Mutation	Human phenotype	Mouse phenotype
AChR deficiency (*CHRNE* null)	Variable weakness but normal lifespan	Die 12–14 weeks [18, 19, 20]
RAPSN‐CMS p.Asn88Lys (c.264C>A)	Reduced fetal movement Neonatal episodic apnea By adulthood only mild weakness	Die at birth [21]
DOK7‐CMS 1124_1127dupTGCC	Variable limb‐girdle muscle weakness	Die between 12 to 20 days after birth [22, 23]

## 3 AChR Deficiency due to Mutations in CHRNE

The most common CMS is as an autosomal recessive disorder in which mutations in CHRNE (the AChR ε‐subunit) cause a primary deficiency of AChR at the endplate [1, 8, 24, 25]. The phenotype may vary from mild to severe [8, 26]. Weakness is usually evident at birth or within the first year of life and is characterized by feeding difficulties, ptosis, impaired eye movements, and delayed motor milestones. The impaired eye movements are not always apparent in the early neonate and may only develop during the first year. Patients sometimes show improvement in adolescence, and in general, the disease is not progressive. Patients improve with anticholinesterase medication or 3,4‐diaminopyridine, and a clinical history of improvement with these treatments can point to the underlying genetic cause. More recently, the addition of β2‐adrenergic receptor agonists has also been demonstrated as beneficial [26]. Three Hz repetitive nerve stimulation typically shows a decrement of CMAPs, and single‐fiber EMG shows increased jitter and blocking. Intracellular microelectrode recordings from the endplate region of biopsied muscle show that miniature end‐plate potentials (MEPPs) and miniature end‐plate currents (MEPCs) may be decreased compared to normal values. Consistent with MEPP and MEPC findings, staining with ^125^I‐α‐bungarotoxin or alexofluor‐conjugated α‐bungarotoxin show reduced numbers of AChR that are often distributed abnormally along the muscle fiber [27]. In addition, electron microscopy shows a reduction of the postsynaptic folds.

Mutations that give rise to this CMS are located along the length of the ε‐subunit gene and may cause a frameshift and premature termination of translation, affect the promoter [28] or the signal peptide, or affect peptide folding and assembly of the AChR pentamer. Many are null mutations [24, 25]. In these, the residual expression of the γ or fetal subunit that is incorporated at low levels into the endplate receptors, is able to mediate synaptic transmission, and thus partially compensate for loss of adult AChR. Evidence to support this hypothesis includes recordings from endplates that demonstrate the receptors have functional properties of fetal AChR [24], that γ‐subunit mRNA is present at motor endplates of adult AChR deficiency patients [11], and that an animal model in which the adult AChR is replaced by fetal AChR mimics the human condition [29]. The partial compensation through residual γ‐subunit expression explains why the majority of AChR mutations are located within the *CHRNE* locus, since the human endplate can adapt to loss of the ε‐subunit. Many appear as “private” mutations, but founder effects are evident for a number of mutations within European populations. In particular, the mutation ε1327delG [30], which tends to manifest a relatively mild phenotype, is common in Southeastern Europe [31]. The underlying basis for the difference between mild and severe AChR deficiency phenotypes has not been determined but potential explanations are associated with the level and location of the γ‐subunit expression or interference in assembly of the AChR pentamer by the defective mutated ε‐subunit. In situ hybridization demonstrated high levels of mutated ε‐subunit mRNA at the endplates of AChR deficiency patients [11]. Mutations in *CHRNA1*, *CHRNB1*, and *CHRND* underlying AChR deficiency often cause a severe phenotype probably because they cannot be compensated by expression of an alternative subunit, however, there are examples of mutations in each of these subunits that give rise to CMS with a mild phenotype. Mutations that underlie AChR deficiency may also change the kinetics or functional properties of the AChR [32]. In these cases the primary pathogenic defect is loss of endplate AchR, and the altered channel kinetics have a secondary effect. However, if the numbers of endplate AChR are not severely reduced, the phenotype is determined by the altered ion channel kinetics.

## 4 AChR Deficiency due to RAPSN Mutations

Mutations in the AChR‐clustering protein RAPSN also cause a deficiency of endplate AChR [33]. However, the phenotype of these patients is distinct from both AChR deficiency [34] syndromes and syndromes caused by mutations in the AGRN‐LRP4‐MUSK‐DOK7 AChR‐clustering pathway and therefore is categorized as a distinct CMS subset. Onset of manifestations is usually at birth (early onset) although occasional late onset patients presenting from early adulthood through to middle age have been reported [35]. Early onset cases are frequently associated with hypotonia, marked bulbar dysfunction often necessitating nasogastric feeding and may require assisted ventilation. Slight facial malformation, a high arched palate and joint contractures of hands and ankles are common. Although eye movement is usually normal, strabismus is frequent. In childhood the course of disease is associated with severe exacerbations often presenting with life‐threatening respiratory failure. Patients tend to improve over time, severe apneic episodes are rarer after the age of 6 years, and in many cases in adulthood disability is minimal. Late onset patients may be mistaken for seronegative immune‐mediated myasthenia gravis. Weakness of ankle dorsiflexion, which is uncommon in myasthenia gravis, may provide a clue that RAPSN mutations underlie the condition [35]. Both early and late onset cases show an abnormal decrement of the CMAP on slow (3 Hz) repetitive nerve stimulation and jitter on single fiber EMG, although it is not always easy to detect. Patients with *RAPSN* mutations respond well to anticholinesterase medication and may gain further benefit from the addition of 3,4‐diaminopyridine.

On muscle biopsy, endplates from patients with AChR deficiency due to *CHRNE* mutations or AChR deficiency from *RAPSN* mutations appear similar [33]; however, the two conditions are clearly clinically distinct. The underlying cause of these differential features is unclear. However, whereas patients with ε‐subunit null mutations most likely survive through maintained low‐level expression of the fetal (γ) subunit, patients with *RAPSN* mutations express the ε‐subunit. Thus, one clear difference in mature muscle between the *RAPSN* group and *CHRNE* group is the type of endplate AChR that mediates the synaptic transmission.

As is the case for many CMS, RAPSN‐CMS show recessive inheritance [33, 34, 35, 36, 37, 38, 39, 40, 41]. RAPSN proved difficult to purify and crystallize and so, thus far, knowledge about its structure is derived from molecular modeling that needs verification. Mutations are observed along the length of the protein but there are no clear phenotypic associations with their positions within RAPSN. However, most patients harbor the missense mutation p.Asn88Lys (N88K) on at least one allele, suggesting an original founder mutation [36]. The frequency of RAPSN‐N88K suggests that it retains at least partial function which is supported by cell culture experiments, in which RAPSN‐N88K was able to mediate AGRN‐induced AChR clusters but these clusters were found to be less stable than clusters formed with wild type rapsyn [40]. Many other variants were found to drastically inhibit RAPSN function and AChR/RAPSN interaction and therefore are null mutants [40]. Thus, it may be that in patients with the N88K mutation AChR deficiency is due to instability of the endplate RAPSN‐N88K/AChR clusters. The observation that RAPSN‐CMS patients tend to improve over time and often show minimal weakness in adulthood would indicate that the structure of the neuromuscular junction once fully formed is sufficient to hold the AChR in place in the membrane without the full anchoring by RAPSN.

Most patients with AChR deficiency due to RAPSN mutations harbor the N88K mutation on at least one allele. In the first description of this disorder, all four reported cases harbored N88K [33] and in a follow‐up report of 39 cases, all but those homozygous for a promoter mutation harbored N88K on at least one allele [41]. Rarely, patients are heteroallelic or homozygous for mutations in the coding region that are not N88K. A number of patients have been identified with mutations in the promoter region of the RAPSN gene. Some of these promoter mutations severely reduce rapsyn mRNA transcription, but others, such as −38A>G, have a less drastic effect on transcription, and this mutation has been found homozygous in patients of Iranian Jewish origin who commonly show facial malformation [42]. The facial malformation, high‐arched palate, and joint contractures observed in patients with RAPSN mutations are all thought to result from lack of fetal movement, presumably due to failed neuromuscular transmission at critical stages in fetal development. Thus, the RAPSN mutations must affect clustering of both the adult and the fetal AChR subtypes.

## 5 Congenital Myasthenic Syndromes due to Mutations in DOK7

Mutations in the AGRN‐LRPR‐MUSK‐DOK7 AChR clustering pathway give rise to CMS with characteristic “limb girdle” pattern of myasthenic muscle weakness. Mutations in DOK7 are by far the most common of these [8, 16, 43, 44, 45]. Clinical onset of disease is generally characterized by difficulty in walking after initial achievement of walking milestones. Patients occasionally have earlier signs of ptosis, floppy tone, bulbar, and respiratory problems. The walking and running impairment tends to worsen in childhood and is accompanied by upper limb weakness and loss of ambulation in some patients. A waddling and lordotic gait is often seen associated with proximal lower limb and truncal weakness. Ptosis is often present from an early age, though it may develop and progress in childhood. Eye movements are usually normal, while facial, jaw, and neck weakness are common and tongue wasting has been observed in adulthood in around 50% of patients. Fluctations in symptoms over weeks or months are common. In many cases, diagnosis as a myasthenic disorder may be delayed, and disorders such as muscular dystrophy and congenital myopathy suggested [43, 44]. There is a remarkable improvement with β2 adrenergic receptor agonists, ephedrine, and salbutamol [43, 44, 45, 46, 47, 48]. However, contrasting with the characteristic myasthenic response to pyridostigmine or 3,4‐DAP which usually is felt within an hour, the response to β2 adrenergic receptor agonists occurs gradually over weeks to months, and it can take up to 2 years before the full benefit is achieved. Features seen in patients with *RAPSN* mutations such as congenital joint deformities and weakness of ankle dorsiflexion, which are associated with reduced fetal movement in the womb, have not been observed.

When the *DOK7* gene was “knocked out” in mice, neuromuscular junctions failed to form and offspring failed to survive past birth [49]. Splice site, missense, and frameshift mutations have been identified in *DOK7* [50], and recessive inheritance of these results in a myasthenic syndrome with the characteristic proximal or “limb girdle”‐type weakness described above. The majority of the mutations are located in the large 3′ exon of the gene that encodes the C‐terminal region of DOK7. Mutation 1124_1127dupTGCC is common. It is thought that DOK7 activates MuSK through a DOK7 phosphotyrosine binding domain interacting with the juxtamembrane phosphotyrosine binding motif of MuSK, leading to dimerisation and formation of the AGRN‐LRP4‐MUSK‐DOK7 complex in the postsynaptic membrane. This binding can occur even when the C‐terminal region is truncated [16]. Studies of the motor endplates from patients harboring DOK7 mutations found that components of the neuromuscular junction were present at normal density and showed normal function but that the sizes of the pre‐ and postsynaptic structures were much smaller than normal [16, 17, 45]. In the initial study of patients with DOK7 mutations, all (*n* = 19) showed decrements of CMAP at 3 Hz stimulation and/or increased jitter and blocking on SFEMG [16, 17] In addition, there is evidence for the synaptic structure degenerating and reforming [45]. These observations, in combination with functional studies of the action of mutant DOK7 on the AChR‐clustering pathway, indicate that the major effect of DOK7 mutations is on impaired maturation and maintenance of neuromuscular junction structure.

## 6 Summary

The key clinical features of these three forms of CMS are outlined in Table 2. For CHRNE null mutations, the low level of γ‐subunit expression maintained throughout life enables survival; in RAPSN‐CMS, AChR clusters form but are unstable; and for DOK7‐CMS, maintenance of the structural integrity of the neuromuscular synapse is compromised. Though in all cases synaptic transmission is impaired, these differing underlying pathogenic mechanisms lead to divergent phenotypic characteristics. The contrasting relative mild severity of the human condition compared to mouse models suggests multiple facets of the human neuromuscular junction that are yet to be understood, but that the plasticity of this synapse that can respond to treatments even late in life augurs well for the efficacy of new therapies that are under development.

**TABLE 2:** Distinguishing clinical features of the three most common forms of CMS.

Clinical feature	CHRNE (deficiency)	RAPSN	DOK7
Joint contractures at birth	No	Frequent	Very rare
Ophthalmoplegia	In nearly all cases	Rare	Rare
Strabismus	No	Common	Rare
Episodic crises	Rare	Common	Rare
Tongue wasting	Not reported	Not reported	~50% adults
Pattern of weakness	Generalized	Generalized	Focus on limb girdle
Response to anticholinesterase	Good	Good	Detrimental
Respond 3,4‐diaminopyridine	Good	Good	Modest or detrimental
Response to β‐adrenergic receptors	Good in combination with anticholinesterases	Good in combination with anticholinesterases	Good with improvement over months
Late onset	Rare	Can occur	Can occur
Delayed walking	Common	Common	Rare
Prognosis	Stable	Improves	Stable/gradually worsens

## Author Contributions

**David Beeson:** conceptualization, investigation, funding acquisition, writing – original draft, writing – review and editing.

## Funding

The author has nothing to report.

## Disclosure

I confirm that I have read the Journal's position on issues involved in ethical publication and affirm that this report is consistent with those guidelines.

## Ethics Statement

The author has nothing to report.

## Conflicts of Interest

The author declares no conflicts of interest.

## Data Availability

Data sharing not applicable to this article as no datasets were generated or analyzed during the current study.
